# Small bowel malignant melanoma presenting as a perforated jejunal diverticulum: a case report and literature review

**DOI:** 10.1093/gastro/gou058

**Published:** 2014-08-20

**Authors:** Richard C. Newton, Nicholas Penney, Nicholas Nind, Muhammad S. Sajid, Parvinder Sains

**Affiliations:** ^1^The Department of General Surgery, Worthing Hospital, Western Sussex Hospitals NHS Foundation Trust, Worthing, West Sussex, N11 2DH, UK; ^2^The Department of Histopathology, Worthing Hospital, Western Sussex Hospitals NHS Foundation Trust, Worthing, West Sussex, N11 2DH, UK

**Keywords:** malignant melanoma, intestinal perforation, jejunal diverticulum

## Abstract

Although usually harmless and asymptomatic, jejuno-ileal diverticulae are associated with various non-specific gastrointestinal symptoms, and rarely cause surgical emergencies. This case report describes the presentation and management of a patient with an acute abdomen, whose jejunal diverticulum was perforated. Unexpectedly, histopathological assessment demonstrated malignant melanoma lining the diverticulum. Whether this was primary or metastatic is discussed, together with a synopsis of the literature on small bowel diverticulae.

## INTRODUCTION

Jejuno-ileal diverticulae (JID) are acquired outpouchings of the small bowel, emerging on the mesenteric border. They develop probably as the result of intestinal dyskinesis and high segmental intraluminal pressures. They can be associated with abdominal pain, diarrhoea, or unexplained bleeding, but usually cause no harm. Occasionally they cause a surgical emergency through volvulus, obstruction, or, as in the case described below, through perforation. The precipitant for perforation in this case report was previously undescribed: infiltration of malignant melanoma into the diverticulum.

## CASE PRESENTATION

A 64-year-old Caucasian female presented with a 48-hour history of rapid-onset severe epigastric and right upper quadrant pain after eating fish pie. The pain radiated to the right shoulder, was exacerbated by movement, and was associated with rigors, a couple of episodes of vomiting and some dyspnoea. After a chest infection a few months previously, she had been left with a persistent, non-productive cough that had not responded to courses of antibiotics and steroids. It had lowered her exercise tolerance; where she worked as a manager of sheltered accommodation, she was now using the lift rather than the stairs. She had no other past medical or surgical history and had never smoked.

**Figure 1:**
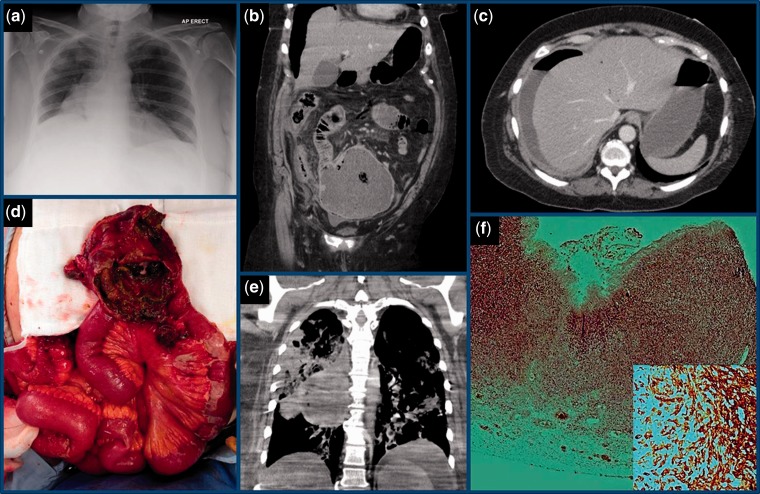
The initial chest X-ray showed a raised right hemidiaphragm but no free air (a). The first CT scan showed a fluid-filled diverticulum continuous with the small bowel (b), along with extraluminal air and fluid, and portal venous air within the left lobe of the liver (c). The mucosal lining of the perforated diverticulum was necrotic and had a brown hue (d). The second CT scan extended into the chest and showed consolidation and a necrotic cystic lesion in the right lung (e). Histopathological assessment showed full-thickness infiltration of tumour across the bowel wall (f), staining for Melan A (inset; high magnification).

On examination in the Accident and Emergency Department her blood pressure was 170/85 mm Hg with SaO_2_ of 97% on room air, but her pulse was 140 beats per minute with a respiratory rate of 38 breaths per minute. Her abdomen was distended, with marked tenderness and guarding in the right upper quadrant, and Murphy’s sign was positive. Abdominal and erect chest radiographs showed a raised right hemidiaphragm (Figure 1a). Venous blood tests showed haemoglobin (Hb) 135 g/L, white blood count (WBC) 12.6 × 10^9^/L, urea 7.8 mmol/L, creatinine 122 μmol/L, amylase 43 U/L, C-reactive protein (CRP) 332 mg/L, and liver function tests within normal range. Arterial blood gases on room air were interpreted as a lactic acidaemia (lactate 4.9 mmol/L, base excess −6.5 mmol/L, and HCO_3_ 19.9 mmol/L) fully compensated by tachypnoea (pH 7.47, pO_2_ 12.7 kPa, pCO_2_ 3.12 kPa). Despite resuscitation with intravenous fluid and antibiotics for a provisional diagnosis of severe acute cholecystitis, she remained oliguric, and her signs of sepsis failed to improve. An urgent contrast enhanced abdominoperineal computed tomography (CT) scan showed extraluminal air and fluid, mild thickening and inflammation of the right colon, several small pockets of portal venous air emboli within the liver, and a large, small-bowel diverticulum within the pelvis containing solid material and air (Figure 1b and c).

A midline laparotomy was performed. Diffuse small bowel contents were causing a generalised peritonitis with adhesions and a widespread fibrinous exudate. A perforated 10 cm diameter jejunal diverticulum, 80 cm from the ligament of Treitz, was found within an abscess cavity (Figure 1d). The diverticulum was gradually freed from several adhesions to more distal small bowel, before resection along with a short section of damaged ileum. The continuity of the small bowel was restored by hand-sewn interrupted seromuscular end-to-end anastamoses using 3/0 Vicryl®. After copious warm saline wash-out, two large-bore surgical drains were inserted and the abdomen was closed with loop nylon. The patient was returned to intensive care but became and remained acidotic, with fast atrial fibrillation, renal failure, chest sepsis and inotrope dependency. Owing to rising inflammatory markers, a repeat CT scan was performed on Day 4, which did not show any formed intra-abdominal collections or evidence of anastamotic leak, but in the lungs there was diffuse, predominantly dependent consolidation and ‘ground glass' opacification. Furthermore, there was a 10 cm multicystic necrotic lesion in the upper segment of the right lower lobe, contiguous with grossly enlarged necrotic paratracheal adenopathy (Figure 1e).

Histopathological examination of the prepared surgical specimen showed an ulcerated necrotic epithelium lining the jejunal diverticulum, with normal small bowel epithelium proximally and distally. Pleomorphic tumour cells had infiltrated the wall of the diverticulum (Figure 1f). The tumour cells had vesicular nuclei and many contained prominent nucleoli. Some cells contained brown pigment that was negative on Perl’s staining for iron/haemosiderin, but strongly expressed the immunohistochemical melanoma markers S100 and Melan A (Figure 1f, inset). This supported a diagnosis of malignant melanoma of primary or metastatic nature within the jejunal diverticulum. The lung lesion was therefore also likely to be malignant melanoma. Notably, there were no suspicious cutaneous or ocular pigmented lesions. The patient continued to deteriorate and, when the prospects of recovery became negligible, after discussion with the family, the patient was palliated and passed away on Day 13.

## DISCUSSION

The prevalence of JID on autopsy ranges between 0.3 and 4.6%, depending on whether the small bowel is distended with gas; with small bowel contrast studies the prevalence is 0.5–2.3% [1–3]. Small antimesenteric hernias may be missed during surgery, due to becoming hidden within the small bowel mesentery. Whilst JIDs are asymptomatic in the majority of patients, they are sometimes linked with (i) vague abdominal symptoms and signs such as intermittent pains, diarrhoea, malabsorption and steattorhoea, (ii) flatulence from small bowel bacterial overgrowth, (iii) bleeding and anaemia (pre-operative localisation of the bleeding is often delayed, especially as half of patients with JID have co-existing colonic diverticulae), (iv) megaloblastic anaemia secondary to the intraluminal metabolism of B12 from bile acid deconjugation from coliform overgrowth due to stasis, (v) obstruction (due to volvulus, enterolith formation or adhesions owing to previous bouts of jejuno-ileal diverticulutis), and (vi) perforation (this can be localised or cause widespread peritonitis). Perforation of a JID usually mandates a laparotomy and carries a risk of mortality of 20–40% [4]. Our patient’s initial risk of mortality was probably greater, owing to signs of severe sepsis and the poor prognostic radiological finding of hepatic portal venous air [5]. Interestingly, analogous to colonic diverticulitis, if the patient is well and the signs minimal, conservative management has been successfully employed [6].

Small bowel diverticulae are diagnosed with abdominal radiographs (multiple air fluid levels), enteroclysis (outpouching with retained contrast after the main lumen has emptied), CT, CT enteroclysis, or at laparoscopy/laparotomy. The mean age at diagnosis is 70 [7]. Small bowel diverticulae were first described by Baillie and Soemmering in 1794 [8], Sir Astley Cooper described specifically jejunal diverticulae in 1807 [9], Sir William Osler reported the condition causing fatal malaena in 1881 [10] and, in 1906, Gordinier and Sampson described emergency surgical management [11]. Unlike Meckel’s, which are antimesenteric, congenital and true diverticulae, most JID are believed to be acquired pulsion lesions, and therefore false diverticulae, comprising a thin wall lacking a muscle coat. They are usually found on the mesenteric aspect where the *vasa recta* (paired blood vessels) penetrate and thus weaken the bowel wall [12]; small bowel diverticulae are both more common and more likely to be multiple within the jejunum, where feeding vessels have the greatest diameter. Abnormal neuromuscular contractility has been proposed as an aetiological factor [13], and steroids and non-steroidal anti-inflammatory drugs have also been implicated [14]. There is an association between small bowel diverticulae and colonic diverticulae, suggesting connective tissue or dietary factors.

Primary malignancies of the small bowel are rare, and more commonly found in the jejunum than the ileum, but primary adenocarcinomas are the most common, followed by carcinoids, lymphomas and stromal tumours [15]. Small bowel melanoma is usually metastatic, arising from the skin more often than the eye or anus; indeed, although neural crest cells embryologically migrate into the gut, mature melanocytes are a rare entity in the small bowel, so there is controversy as to whether small bowel melanoma can ever be considered primary, with some authors suggesting that an occult or regressed primary is always the original source (spontaneous regression of cutaneous melanomas can occur) [16]. Nevertheless, there are nearly 30 case reports on PubMed® claiming a diagnosis of primary gastrointestinal malignant melanoma, but the follow-up after resection is often short. Evidence that would support a diagnosis of primary melanoma includes the pathological identification, in the surrounding tissue, of melanosis or a precursor lesion, a long disease-free survival period after resection [17], and absence of extraintestinal metastatic spread. The case described here matches none of these and, without the potential help of a post-mortem, it therefore more likely represents metastatic cutaneous melanoma to bowel and lung. In a post-mortem study of 216 patients with advanced melanoma, multiple organ deposits were present in 95% of patients, with the organs most commonly involved being lymph nodes (74%), lungs (71%), liver (58%), brain (49%), bone (49%), heart (47%), adrenal glands (47%), and gastrointestinal tract (44%) [18]. The normal clinical presentation of gastrointestinal melanoma is similar to other malignancies: a change in bowel habit or obstructive symptoms, a palpable mass, rectal bleeding or anaemia and fatigue, and pain [19]. Presentation via a perforated JID has not been previously described.

Many different primary cancers have been infrequently reported in cases of Meckel’s diverticulae: adenocarcinomas, stromal tumours, sarcomas, neuroendocrine tumours [20], and malignant melanoma have been detected [21]. It has even been controversially proposed that incidentally discovered Meckel’s should be resected, owing to their carrying a relatively high risk of cancer [22]. Jejunal diverticulae have contained sarcomas [23], stromal tumours [24], and leiomyosarcomas [25], but this is the first reported case of one containing malignant melanoma.

**Conflict of interest:** none declared.
